# 4-(4,5-Diphenyl-1*H*-imidazol-2-yl)-*N*,*N*-di­methyl­aniline

**DOI:** 10.1107/S160053681301444X

**Published:** 2013-05-08

**Authors:** M. Prabhuswamy, S. Viveka, S. Madan Kumar, G. K. Nagaraja, N. K. Lokanath

**Affiliations:** a Department of Studies in Physics, University of Mysore, Mysore 570 006, India; b Department of Chemistry, Mangalore University, Mangalore 574 199, India

## Abstract

The asymmetric unit of the title compound, C_23_H_21_N_3_, consists of two symmetry-independent and conformationally different mol­ecules [the comparable dihedral angles between the imidazole ring and the three benzene rings being 38.5 (2)/61.5 (3)/3.37 (17) and 45.8 (2)/36.01 (19)/46.94 (17)°]. In the crystal, inter­molecular imidazole N—H⋯N hydrogen-bonding inter­actions give a one-dimensional chain extending along [101].

## Related literature

For background on imidazoles, see: Ucucu *et al.* (2001 ▶). For similar structures, see: Yanover & Kaftory (2009 ▶); Akkurt *et al.* (2013 ▶); Prabhuswamy *et al.* (2013 ▶).
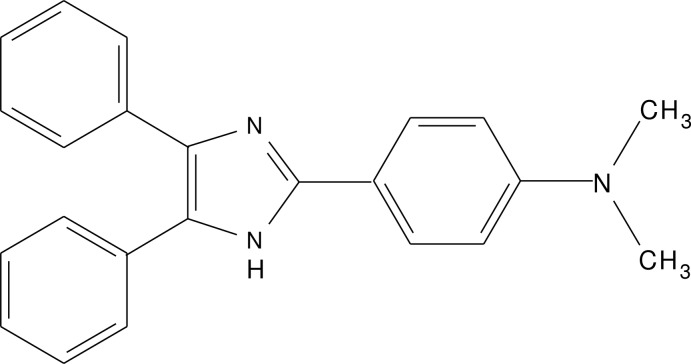


## Experimental

### 

#### Crystal data

C_23_H_21_N_3_
*M* *_r_* = 339.43
Monoclinic, 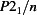

*a* = 15.228 (4) Å
*b* = 15.215 (4) Å
*c* = 17.641 (4) Å
β = 110.974 (4)°
*V* = 3816.5 (17) Å^3^
*Z* = 8
Mo *K*α radiation
μ = 0.07 mm^−1^
*T* = 296 K
0.24 × 0.19 × 0.17 mm

#### Data collection

Oxford Xcalibur Eos (Nova) CCD diffractometer
36558 measured reflections
6983 independent reflections
3921 reflections with *I* > 2σ(*I*)
*R*_int_ = 0.063

#### Refinement

*R*[*F* ^2^ > 2σ(*F* ^2^)] = 0.070
*wR*(*F* ^2^) = 0.175
*S* = 1.02
6983 reflections
475 parameters
H-atom parameters constrained
Δρ_max_ = 0.22 e Å^−3^
Δρ_min_ = −0.17 e Å^−3^

### 

Data collection: *CrysAlis PRO* (Oxford Diffraction, 2009 ▶); cell refinement: *CrysAlis PRO*; data reduction: *CrysAlis RED*; program(s) used to solve structure: *SHELXS97* (Sheldrick, 2008 ▶); program(s) used to refine structure: *SHELXL97* (Sheldrick, 2008 ▶); molecular graphics: *Mercury* (Macrae *et al.*, 2008 ▶); software used to prepare material for publication: *PLATON* (Spek, 2009 ▶).

## Supplementary Material

Crystal structure: contains datablock(s) global, I. DOI: 10.1107/S160053681301444X/zs2260sup1.cif

Structure factors: contains datablock(s) I. DOI: 10.1107/S160053681301444X/zs2260Isup2.hkl

Supplementary material file. DOI: 10.1107/S160053681301444X/zs2260Isup3.cml

Additional supplementary materials:  crystallographic information; 3D view; checkCIF report

## Figures and Tables

**Table 1:** Hydrogen-bond geometry (Å, °)

*D*—H⋯*A*	*D*—H	H⋯*A*	*D*⋯*A*	*D*—H⋯*A*
N1*B*—H1*B*⋯N1*A* ^i^	1.01	1.92	2.899 (3)	163
N3*A*—H3*A*⋯N3*B*	1.02	1.92	2.890 (3)	157

Symmetry code: (i) 
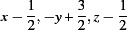
.

**Fig. 1.**
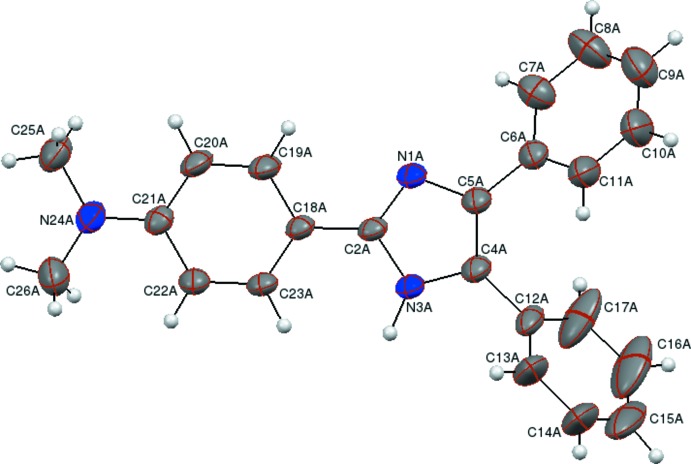
Molecular conformation and atom numbering scheme for molecule A of the title compound showing 30% probability ellipsoids.

**Fig. 2.**
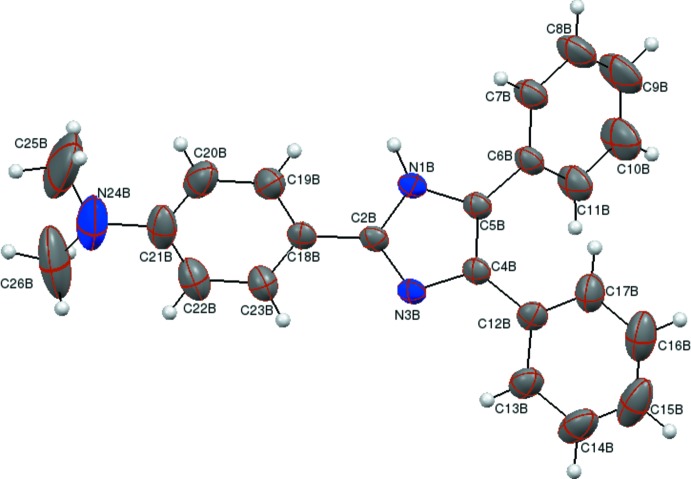
Molecular conformation and atom numbering scheme for molecule B of the title compound showing 30% probability ellipsoids.

**Fig. 3.**
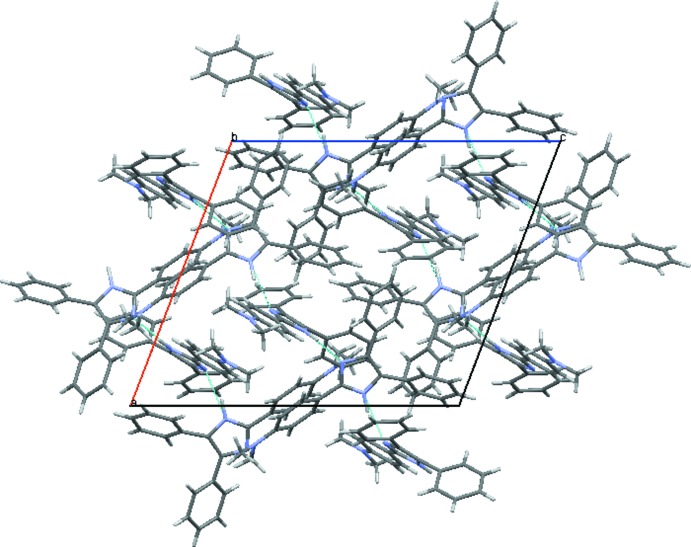
The packing of molecules of the title compound when viewed down the crystallographic b-axis.

## supplementary crystallographic information

### Comment

As a continuation of our studies on the molecular structures of some of the biologically active imidazole derivatives (Ucucu, *et al.*, 2001), we have synthesized the title compound, the substituted imidazole C_23_H_21_N_3_ and the crystal structure is reported herein. The asymmetric unit of this compound consists of two symmetry-independent and conformationally different molecules, *A* and *B* (Fig. 1 & Fig. 2). The two molecules depart significantly from planarity. In molecule *A*, the imidazole ring forms dihedral angles of 38.5 (2), 61.5 (3) and 3.37 (17)° with phenyl rings C6A/C7A/C8A/C9A/C10A/C11A, C12A/C13A/C14A/C15A/C16A/C17A and the dimethylaniline substituted phenyl ring C18A/C19A/C20A/C21A/C22A/C23A respectively. These values compare with 45.8 (2), 36.01 (19) and 46.94 (17)° for the corresponding angles in molecule *B*. The overall geometry of the title compound is similar to that of 4-(1-allyl-4,5-diphenyl-1*H*-imidazol-2-yl)-*N*,*N*- dimethylaniline (Akkurt *et al.*, 2013)

In the crystal, the *A* and *B* molecules are connected by imidazole N—H···N hydrogen bonds (Table 1) both within the asymmetric unit (N1*A*-H···N3*B*) and between the unit (N1*B*-H···N3*B*)^i^, giving chains extending along [1 0 1] (Fig. 3). The crystal structure is also stabilized with short contacts of the type C25B—H25B···*Cg6* [*x* -1/2, *y* + 3/2, *z* +1/2] with a C···*Cg* distance of 3.726 (10) Å (C—H···*Cg* angle, 134°) (where *Cg*6 is C6B/C7B/C8B/C9B/C10B/C11B).

### Experimental

Benzil (1 mmol), *N*,*N*-dimethyl benzaldehyde (1 mmol), and ammonium acetate (2 mmol) were dissolved in boiling glacial acetic acid and refluxed for 5–6 h. The progress of the reaction was monitored by TLC. After completion of the reaction, the reaction mixture was poured into ice-water. The title compound obtained was recrystallized from DMF.

### Refinement

The imidazole N-bound H-atoms (H1B and H3A) were located in a difference Fourier map but were allowed to ride in the refinement with *U*_iso_ = 1.2*U*_eq_(N). All other hydrogen atoms were positioned geometrically and also refined using a riding model with C—H = 0.93–0.96 Å and *U*_iso_(methyl H) = 1.5 *U*_eq_(C) and *U*_iso_(H) = 1.2 *U*_eq_(C) for other hydrogen atoms.

### Crystal data

**Table:**

C_23_H_21_N_3_	*F*(000) = 1440
*M**_r_* = 339.43	*D*_x_ = 1.181 Mg m^−^^3^
Monoclinic, *P*2_1_/*n*	Mo *K*α radiation, λ = 0.71073 Å
Hall symbol: -P 2yn	Cell parameters from 3349 reflections
*a* = 15.228 (4) Å	θ = 1.5–25.4°
*b* = 15.215 (4) Å	µ = 0.07 mm^−^^1^
*c* = 17.641 (4) Å	*T* = 296 K
β = 110.974 (4)°	Block, white
*V* = 3816.5 (17) Å^3^	0.24 × 0.19 × 0.17 mm
*Z* = 8	

### Data collection

**Table:**

Oxford Xcalibur Eos (Nova) CCD diffractometer	*R*_int_ = 0.063
Radiation source: graphite	θ_max_ = 25.4°, θ_min_ = 1.5°
ω scans	*h* = −18→18
36558 measured reflections	*k* = −18→18
6983 independent reflections	*l* = −21→21
3921 reflections with *I* > 2σ(*I*)	

### Refinement

**Table:**

Refinement on *F*^2^	Primary atom site location: structure-invariant direct methods
Least-squares matrix: full	Secondary atom site location: difference Fourier map
*R*[*F*^2^ > 2σ(*F*^2^)] = 0.070	Hydrogen site location: inferred from neighbouring sites
*wR*(*F*^2^) = 0.175	H-atom parameters constrained
*S* = 1.02	*w* = 1/[σ^2^(*F*_o_^2^) + (0.0546*P*)^2^ + 1.5051*P*] where *P* = (*F*_o_^2^ + 2*F*_c_^2^)/3
6983 reflections	(Δ/σ)_max_ < 0.001
475 parameters	Δρ_max_ = 0.22 e Å^−^^3^
0 restraints	Δρ_min_ = −0.17 e Å^−^^3^

### Special details

**Table:**

Geometry. Bond distances, angles *etc*. have been calculated using the rounded fractional coordinates. All su's are estimated from the variances of the (full) variance-covariance matrix. The cell e.s.d.'s are taken into account in the estimation of distances, angles and torsion angles
Refinement. Refinement on *F*^2^ for ALL reflections except those flagged by the user for potential systematic errors. Weighted *R*-factors *wR* and all goodnesses of fit *S* are based on *F*^2^, conventional *R*-factors *R* are based on *F*, with *F* set to zero for negative *F*^2^. The observed criterion of *F*^2^ > σ(*F*^2^) is used only for calculating -*R*-factor-obs *etc*. and is not relevant to the choice of reflections for refinement. *R*-factors based on *F*^2^ are statistically about twice as large as those based on *F*, and *R*-factors based on ALL data will be even larger.

### Fractional atomic coordinates and isotropic or equivalent isotropic displacement parameters (Å^2^)

**Table:**

	*x*	*y*	*z*	*U*_iso_*/*U*_eq_	
N1A	0.86183 (15)	0.76491 (16)	0.18460 (13)	0.0584 (8)	
N3A	0.78361 (16)	0.72591 (16)	0.05759 (13)	0.0602 (8)	
N24A	0.83099 (19)	0.34428 (18)	0.23863 (16)	0.0807 (11)	
C2A	0.82417 (18)	0.6976 (2)	0.13534 (15)	0.0553 (10)	
C4A	0.7950 (2)	0.8155 (2)	0.05703 (17)	0.0673 (11)	
C5A	0.8443 (2)	0.8388 (2)	0.13560 (17)	0.0649 (11)	
C6A	0.8763 (2)	0.9259 (2)	0.1700 (2)	0.0701 (12)	
C7A	0.8746 (2)	0.9490 (3)	0.2452 (2)	0.0911 (17)	
C8A	0.9042 (3)	1.0308 (3)	0.2780 (3)	0.123 (2)	
C9A	0.9386 (4)	1.0895 (3)	0.2368 (4)	0.132 (3)	
C10A	0.9427 (4)	1.0679 (3)	0.1633 (3)	0.133 (2)	
C11A	0.9111 (3)	0.9864 (3)	0.1297 (2)	0.1061 (18)	
C12A	0.7547 (3)	0.8661 (2)	−0.0189 (2)	0.0850 (12)	
C13A	0.7797 (4)	0.8527 (3)	−0.0825 (2)	0.147 (3)	
C14A	0.7401 (7)	0.8992 (4)	−0.1535 (3)	0.222 (5)	
C15A	0.6765 (7)	0.9541 (5)	−0.1633 (4)	0.212 (4)	
C16A	0.6477 (4)	0.9767 (5)	−0.0979 (5)	0.217 (4)	
C17A	0.6869 (3)	0.9276 (4)	−0.0258 (4)	0.170 (3)	
C18A	0.82588 (18)	0.60609 (19)	0.16014 (15)	0.0536 (10)	
C19A	0.8725 (2)	0.5815 (2)	0.24015 (17)	0.0759 (11)	
C20A	0.8746 (2)	0.4964 (2)	0.26631 (18)	0.0757 (11)	
C21A	0.83009 (19)	0.4297 (2)	0.21340 (17)	0.0596 (10)	
C22A	0.7824 (2)	0.4538 (2)	0.13234 (17)	0.0646 (11)	
C23A	0.78093 (19)	0.5392 (2)	0.10748 (16)	0.0597 (10)	
C25A	0.8782 (3)	0.3207 (2)	0.3227 (2)	0.0947 (17)	
C26A	0.7788 (3)	0.2772 (2)	0.1834 (2)	0.1125 (18)	
N1B	0.54844 (15)	0.70966 (15)	−0.19536 (12)	0.0547 (8)	
N3B	0.66562 (15)	0.65014 (15)	−0.09518 (12)	0.0539 (8)	
N24B	0.3533 (3)	0.7156 (4)	0.0775 (2)	0.151 (2)	
C2B	0.58022 (19)	0.68405 (18)	−0.11642 (15)	0.0518 (9)	
C4B	0.68886 (19)	0.65370 (18)	−0.16416 (15)	0.0531 (10)	
C5B	0.61568 (19)	0.68851 (18)	−0.22724 (15)	0.0541 (10)	
C6B	0.5964 (2)	0.6996 (2)	−0.31453 (16)	0.0628 (10)	
C7B	0.5525 (2)	0.7741 (2)	−0.35569 (18)	0.0797 (14)	
C8B	0.5278 (3)	0.7807 (3)	−0.4385 (2)	0.1081 (18)	
C9B	0.5460 (4)	0.7140 (4)	−0.4806 (2)	0.134 (2)	
C10B	0.5898 (4)	0.6390 (3)	−0.4418 (2)	0.127 (2)	
C11B	0.6156 (3)	0.6321 (3)	−0.35784 (19)	0.0899 (15)	
C12B	0.7834 (2)	0.6277 (2)	−0.16052 (16)	0.0622 (10)	
C13B	0.8300 (2)	0.5599 (2)	−0.11106 (19)	0.0844 (14)	
C14B	0.9203 (3)	0.5368 (3)	−0.1059 (2)	0.1113 (19)	
C15B	0.9643 (3)	0.5821 (5)	−0.1483 (3)	0.129 (3)	
C16B	0.9190 (3)	0.6502 (4)	−0.1965 (3)	0.118 (2)	
C17B	0.8294 (3)	0.6736 (3)	−0.2026 (2)	0.0870 (14)	
C18B	0.52508 (19)	0.6943 (2)	−0.06419 (15)	0.0556 (10)	
C19B	0.4782 (2)	0.7711 (3)	−0.06192 (19)	0.0830 (14)	
C20B	0.4225 (3)	0.7786 (3)	−0.0151 (2)	0.1105 (19)	
C21B	0.4111 (3)	0.7093 (4)	0.0308 (2)	0.101 (2)	
C22B	0.4597 (3)	0.6344 (3)	0.0302 (2)	0.0988 (19)	
C23B	0.5161 (2)	0.6276 (2)	−0.01595 (19)	0.0789 (14)	
C25B	0.3008 (6)	0.7926 (7)	0.0707 (5)	0.291 (7)	
C26B	0.3240 (5)	0.6381 (6)	0.1069 (4)	0.213 (4)	
H3A	0.75110	0.68470	0.01040	0.091 (10)*	
H7A	0.85310	0.90870	0.27410	0.1090*	
H8A	0.90080	1.04610	0.32790	0.1480*	
H9A	0.95940	1.14450	0.25920	0.1590*	
H10A	0.96660	1.10770	0.13570	0.1600*	
H11A	0.91330	0.97220	0.07920	0.1270*	
H13A	0.82530	0.81060	−0.07910	0.1760*	
H14A	0.76170	0.88920	−0.19570	0.2670*	
H15A	0.64780	0.98040	−0.21380	0.2550*	
H16A	0.60500	1.02190	−0.10230	0.2610*	
H17A	0.66650	0.93710	0.01730	0.2030*	
H19A	0.90370	0.62450	0.27760	0.0910*	
H20A	0.90650	0.48340	0.32080	0.0910*	
H22A	0.75120	0.41100	0.09470	0.0780*	
H23A	0.74850	0.55280	0.05320	0.0720*	
H25A	0.94410	0.33300	0.33840	0.1420*	
H25B	0.86930	0.25920	0.32970	0.1420*	
H25C	0.85250	0.35420	0.35590	0.1420*	
H26A	0.71400	0.29440	0.15990	0.1690*	
H26B	0.78360	0.22290	0.21220	0.1690*	
H26C	0.80400	0.26970	0.14110	0.1690*	
H1B	0.48000	0.72160	−0.22770	0.089 (10)*	
H7B	0.53950	0.82050	−0.32700	0.0960*	
H8B	0.49850	0.83130	−0.46530	0.1290*	
H9B	0.52880	0.71860	−0.53660	0.1610*	
H10B	0.60220	0.59320	−0.47140	0.1520*	
H11B	0.64570	0.58170	−0.33120	0.1080*	
H13B	0.80100	0.52930	−0.08080	0.1010*	
H14B	0.95080	0.48990	−0.07320	0.1330*	
H15B	1.02480	0.56670	−0.14440	0.1540*	
H16B	0.94900	0.68140	−0.22570	0.1420*	
H17B	0.79960	0.72070	−0.23530	0.1040*	
H19B	0.48410	0.81890	−0.09250	0.0990*	
H20B	0.39220	0.83150	−0.01460	0.1320*	
H22B	0.45490	0.58680	0.06150	0.1180*	
H23B	0.54910	0.57560	−0.01400	0.0950*	
H25D	0.34170	0.84270	0.07980	0.4370*	
H25E	0.27110	0.79200	0.11040	0.4370*	
H25F	0.25360	0.79600	0.01730	0.4370*	
H26D	0.28280	0.65360	0.13510	0.3190*	
H26E	0.37810	0.60800	0.14330	0.3190*	
H26F	0.29140	0.60030	0.06210	0.3190*	

### Atomic displacement parameters (Å^2^)

**Table:**

	*U*^11^	*U*^22^	*U*^33^	*U*^12^	*U*^13^	*U*^23^
N1A	0.0532 (14)	0.0640 (16)	0.0460 (13)	−0.0046 (12)	0.0032 (11)	0.0008 (12)
N3A	0.0611 (15)	0.0609 (16)	0.0428 (13)	−0.0062 (12)	−0.0006 (11)	0.0035 (12)
N24A	0.0814 (19)	0.0698 (19)	0.0711 (18)	−0.0036 (15)	0.0032 (14)	0.0151 (15)
C2A	0.0485 (16)	0.066 (2)	0.0386 (15)	−0.0036 (14)	0.0001 (12)	0.0038 (14)
C4A	0.0624 (19)	0.066 (2)	0.0522 (18)	−0.0049 (16)	−0.0054 (14)	0.0077 (15)
C5A	0.0607 (19)	0.062 (2)	0.0543 (18)	−0.0029 (15)	−0.0011 (14)	0.0027 (16)
C6A	0.065 (2)	0.063 (2)	0.065 (2)	0.0023 (16)	0.0021 (16)	0.0031 (18)
C7A	0.083 (3)	0.092 (3)	0.095 (3)	−0.010 (2)	0.028 (2)	−0.022 (2)
C8A	0.130 (4)	0.102 (4)	0.134 (4)	−0.013 (3)	0.045 (3)	−0.051 (3)
C9A	0.158 (5)	0.079 (3)	0.138 (5)	−0.013 (3)	0.026 (4)	−0.022 (3)
C10A	0.199 (5)	0.074 (3)	0.102 (3)	−0.030 (3)	0.024 (3)	0.002 (3)
C11A	0.146 (4)	0.077 (3)	0.070 (2)	−0.020 (3)	0.008 (2)	0.005 (2)
C12A	0.085 (2)	0.066 (2)	0.065 (2)	−0.0176 (19)	−0.0205 (18)	0.0201 (18)
C13A	0.268 (7)	0.094 (3)	0.052 (2)	0.018 (4)	0.025 (3)	0.018 (2)
C14A	0.431 (13)	0.107 (5)	0.057 (3)	0.003 (6)	0.000 (5)	0.026 (3)
C15A	0.265 (10)	0.138 (6)	0.102 (5)	−0.072 (6)	−0.095 (6)	0.047 (5)
C16A	0.135 (5)	0.174 (7)	0.248 (9)	0.009 (4)	−0.047 (6)	0.118 (7)
C17A	0.091 (3)	0.194 (5)	0.196 (5)	0.038 (4)	0.018 (3)	0.126 (5)
C18A	0.0471 (16)	0.0626 (19)	0.0405 (15)	−0.0011 (13)	0.0027 (12)	0.0046 (14)
C19A	0.082 (2)	0.079 (2)	0.0440 (17)	−0.0184 (18)	−0.0049 (15)	0.0038 (16)
C20A	0.077 (2)	0.085 (2)	0.0441 (17)	−0.0094 (18)	−0.0039 (15)	0.0151 (17)
C21A	0.0459 (16)	0.067 (2)	0.0572 (18)	0.0017 (14)	0.0079 (14)	0.0087 (16)
C22A	0.0607 (19)	0.066 (2)	0.0529 (18)	−0.0048 (15)	0.0029 (14)	−0.0022 (15)
C23A	0.0582 (18)	0.069 (2)	0.0383 (15)	−0.0009 (15)	0.0009 (13)	0.0006 (14)
C25A	0.096 (3)	0.092 (3)	0.083 (3)	0.007 (2)	0.016 (2)	0.028 (2)
C26A	0.132 (4)	0.065 (2)	0.112 (3)	−0.005 (2)	0.009 (3)	0.004 (2)
N1B	0.0489 (14)	0.0685 (16)	0.0374 (12)	0.0035 (11)	0.0040 (11)	0.0005 (11)
N3B	0.0467 (14)	0.0700 (16)	0.0364 (12)	0.0005 (12)	0.0044 (10)	0.0031 (11)
N24B	0.117 (3)	0.249 (6)	0.111 (3)	−0.017 (4)	0.069 (3)	−0.042 (3)
C2B	0.0479 (16)	0.0624 (18)	0.0357 (14)	−0.0022 (14)	0.0037 (12)	−0.0005 (13)
C4B	0.0498 (17)	0.0654 (18)	0.0381 (15)	−0.0025 (14)	0.0086 (12)	−0.0022 (13)
C5B	0.0524 (17)	0.0644 (19)	0.0394 (15)	−0.0021 (14)	0.0090 (13)	−0.0008 (13)
C6B	0.0657 (19)	0.077 (2)	0.0389 (15)	−0.0007 (16)	0.0106 (14)	0.0046 (15)
C7B	0.083 (2)	0.096 (3)	0.0510 (19)	0.004 (2)	0.0130 (17)	0.0125 (18)
C8B	0.126 (3)	0.130 (4)	0.054 (2)	0.011 (3)	0.015 (2)	0.025 (2)
C9B	0.178 (5)	0.164 (5)	0.045 (2)	0.010 (4)	0.022 (3)	0.009 (3)
C10B	0.177 (5)	0.144 (4)	0.055 (2)	0.015 (4)	0.037 (3)	−0.020 (3)
C11B	0.116 (3)	0.101 (3)	0.0478 (19)	0.004 (2)	0.0233 (19)	−0.0087 (19)
C12B	0.0499 (17)	0.082 (2)	0.0433 (16)	0.0009 (16)	0.0029 (14)	−0.0090 (15)
C13B	0.066 (2)	0.113 (3)	0.062 (2)	0.021 (2)	0.0082 (17)	−0.004 (2)
C14B	0.079 (3)	0.158 (4)	0.075 (3)	0.045 (3)	0.001 (2)	−0.019 (3)
C15B	0.065 (3)	0.213 (6)	0.099 (4)	0.009 (4)	0.019 (3)	−0.070 (4)
C16B	0.082 (3)	0.177 (5)	0.108 (4)	−0.029 (3)	0.049 (3)	−0.046 (3)
C17B	0.072 (2)	0.114 (3)	0.084 (2)	−0.009 (2)	0.039 (2)	−0.009 (2)
C18B	0.0470 (16)	0.073 (2)	0.0371 (15)	−0.0029 (15)	0.0031 (12)	−0.0041 (14)
C19B	0.088 (2)	0.103 (3)	0.056 (2)	0.020 (2)	0.0235 (19)	−0.0014 (18)
C20B	0.094 (3)	0.160 (4)	0.074 (3)	0.041 (3)	0.026 (2)	−0.017 (3)
C21B	0.073 (3)	0.172 (5)	0.060 (2)	−0.020 (3)	0.025 (2)	−0.032 (3)
C22B	0.105 (3)	0.126 (4)	0.075 (3)	−0.031 (3)	0.044 (2)	−0.005 (2)
C23B	0.088 (2)	0.089 (3)	0.066 (2)	−0.004 (2)	0.0353 (19)	0.0015 (19)
C25B	0.220 (8)	0.500 (17)	0.201 (8)	0.127 (10)	0.133 (7)	−0.049 (9)
C26B	0.181 (6)	0.353 (11)	0.154 (5)	−0.121 (7)	0.121 (5)	−0.066 (6)

### Geometric parameters (Å, º)

**Table:**

N1A—C2A	1.333 (4)	C22A—H22A	0.9300
N1A—C5A	1.385 (4)	C23A—H23A	0.9300
N3A—C2A	1.357 (3)	C25A—H25A	0.9600
N3A—C4A	1.375 (4)	C25A—H25C	0.9600
N24A—C21A	1.372 (4)	C25A—H25B	0.9600
N24A—C25A	1.443 (4)	C26A—H26A	0.9600
N24A—C26A	1.438 (4)	C26A—H26C	0.9600
N3A—H3A	1.0200	C26A—H26B	0.9600
N1B—C2B	1.358 (3)	C2B—C18B	1.460 (4)
N1B—C5B	1.371 (4)	C4B—C12B	1.472 (4)
N3B—C4B	1.385 (3)	C4B—C5B	1.369 (4)
N3B—C2B	1.323 (4)	C5B—C6B	1.471 (4)
N24B—C21B	1.408 (6)	C6B—C11B	1.373 (5)
N24B—C26B	1.423 (10)	C6B—C7B	1.383 (4)
N24B—C25B	1.399 (12)	C7B—C8B	1.376 (4)
N1B—H1B	1.0100	C8B—C9B	1.344 (7)
C2A—C18A	1.457 (4)	C9B—C10B	1.374 (7)
C4A—C12A	1.475 (4)	C10B—C11B	1.394 (5)
C4A—C5A	1.366 (4)	C12B—C13B	1.373 (4)
C5A—C6A	1.466 (4)	C12B—C17B	1.379 (5)
C6A—C11A	1.380 (5)	C13B—C14B	1.390 (6)
C6A—C7A	1.382 (5)	C14B—C15B	1.357 (7)
C7A—C8A	1.379 (6)	C15B—C16B	1.361 (9)
C8A—C9A	1.369 (8)	C16B—C17B	1.377 (7)
C9A—C10A	1.361 (8)	C18B—C23B	1.362 (4)
C10A—C11A	1.384 (6)	C18B—C19B	1.377 (5)
C12A—C17A	1.366 (7)	C19B—C20B	1.384 (5)
C12A—C13A	1.323 (6)	C20B—C21B	1.378 (7)
C13A—C14A	1.376 (7)	C21B—C22B	1.361 (7)
C14A—C15A	1.243 (13)	C22B—C23B	1.383 (5)
C15A—C16A	1.416 (11)	C7B—H7B	0.9300
C16A—C17A	1.410 (10)	C8B—H8B	0.9300
C18A—C19A	1.385 (4)	C9B—H9B	0.9300
C18A—C23A	1.383 (4)	C10B—H10B	0.9300
C19A—C20A	1.371 (4)	C11B—H11B	0.9300
C20A—C21A	1.381 (4)	C13B—H13B	0.9300
C21A—C22A	1.401 (4)	C14B—H14B	0.9300
C22A—C23A	1.369 (4)	C15B—H15B	0.9300
C7A—H7A	0.9300	C16B—H16B	0.9300
C8A—H8A	0.9300	C17B—H17B	0.9300
C9A—H9A	0.9300	C19B—H19B	0.9300
C10A—H10A	0.9300	C20B—H20B	0.9300
C11A—H11A	0.9300	C22B—H22B	0.9300
C13A—H13A	0.9300	C23B—H23B	0.9300
C14A—H14A	0.9300	C25B—H25D	0.9600
C15A—H15A	0.9300	C25B—H25E	0.9600
C16A—H16A	0.9300	C25B—H25F	0.9600
C17A—H17A	0.9300	C26B—H26D	0.9600
C19A—H19A	0.9300	C26B—H26E	0.9600
C20A—H20A	0.9300	C26B—H26F	0.9600
			
C2A—N1A—C5A	106.0 (2)	H25B—C25A—H25C	109.00
C2A—N3A—C4A	108.0 (2)	H26A—C26A—H26C	109.00
C21A—N24A—C25A	120.8 (3)	H26B—C26A—H26C	109.00
C21A—N24A—C26A	120.9 (3)	N24A—C26A—H26C	109.00
C25A—N24A—C26A	118.1 (3)	H26A—C26A—H26B	110.00
C2A—N3A—H3A	123.00	N24A—C26A—H26A	109.00
C4A—N3A—H3A	129.00	N24A—C26A—H26B	109.00
C2B—N1B—C5B	108.0 (2)	N1B—C2B—N3B	110.9 (2)
C2B—N3B—C4B	105.7 (2)	N1B—C2B—C18B	122.8 (3)
C25B—N24B—C26B	118.8 (6)	N3B—C2B—C18B	126.3 (2)
C21B—N24B—C26B	120.0 (6)	N3B—C4B—C5B	109.9 (3)
C21B—N24B—C25B	117.7 (6)	C5B—C4B—C12B	129.7 (3)
C5B—N1B—H1B	126.00	N3B—C4B—C12B	120.3 (2)
C2B—N1B—H1B	123.00	C4B—C5B—C6B	134.0 (3)
N1A—C2A—N3A	110.4 (3)	N1B—C5B—C6B	120.3 (2)
N3A—C2A—C18A	124.0 (2)	N1B—C5B—C4B	105.5 (2)
N1A—C2A—C18A	125.5 (2)	C5B—C6B—C7B	121.5 (3)
N3A—C4A—C5A	106.0 (2)	C5B—C6B—C11B	119.6 (3)
C5A—C4A—C12A	133.2 (3)	C7B—C6B—C11B	118.7 (3)
N3A—C4A—C12A	120.8 (3)	C6B—C7B—C8B	121.0 (3)
N1A—C5A—C6A	120.9 (3)	C7B—C8B—C9B	120.0 (4)
N1A—C5A—C4A	109.6 (3)	C8B—C9B—C10B	120.7 (3)
C4A—C5A—C6A	129.4 (3)	C9B—C10B—C11B	119.6 (4)
C7A—C6A—C11A	117.9 (3)	C6B—C11B—C10B	120.0 (4)
C5A—C6A—C11A	121.5 (3)	C13B—C12B—C17B	118.4 (3)
C5A—C6A—C7A	120.6 (3)	C4B—C12B—C13B	120.3 (3)
C6A—C7A—C8A	121.1 (4)	C4B—C12B—C17B	121.2 (3)
C7A—C8A—C9A	119.7 (5)	C12B—C13B—C14B	120.4 (3)
C8A—C9A—C10A	120.4 (5)	C13B—C14B—C15B	120.5 (4)
C9A—C10A—C11A	119.8 (5)	C14B—C15B—C16B	119.4 (5)
C6A—C11A—C10A	121.1 (4)	C15B—C16B—C17B	120.8 (5)
C13A—C12A—C17A	117.9 (4)	C12B—C17B—C16B	120.5 (4)
C4A—C12A—C13A	122.5 (4)	C19B—C18B—C23B	116.4 (3)
C4A—C12A—C17A	119.6 (4)	C2B—C18B—C19B	122.0 (3)
C12A—C13A—C14A	121.8 (6)	C2B—C18B—C23B	121.6 (3)
C13A—C14A—C15A	122.5 (7)	C18B—C19B—C20B	121.5 (4)
C14A—C15A—C16A	120.2 (7)	C19B—C20B—C21B	121.4 (4)
C15A—C16A—C17A	116.9 (6)	N24B—C21B—C22B	121.2 (5)
C12A—C17A—C16A	120.4 (5)	N24B—C21B—C20B	121.9 (5)
C19A—C18A—C23A	115.8 (3)	C20B—C21B—C22B	116.9 (4)
C2A—C18A—C23A	123.4 (2)	C21B—C22B—C23B	121.4 (4)
C2A—C18A—C19A	120.8 (2)	C18B—C23B—C22B	122.3 (3)
C18A—C19A—C20A	122.7 (3)	C6B—C7B—H7B	120.00
C19A—C20A—C21A	121.3 (3)	C8B—C7B—H7B	119.00
C20A—C21A—C22A	116.6 (3)	C7B—C8B—H8B	120.00
N24A—C21A—C20A	122.1 (3)	C9B—C8B—H8B	120.00
N24A—C21A—C22A	121.4 (3)	C8B—C9B—H9B	120.00
C21A—C22A—C23A	121.2 (3)	C10B—C9B—H9B	120.00
C18A—C23A—C22A	122.4 (3)	C9B—C10B—H10B	120.00
C8A—C7A—H7A	119.00	C11B—C10B—H10B	120.00
C6A—C7A—H7A	119.00	C6B—C11B—H11B	120.00
C9A—C8A—H8A	120.00	C10B—C11B—H11B	120.00
C7A—C8A—H8A	120.00	C12B—C13B—H13B	120.00
C10A—C9A—H9A	120.00	C14B—C13B—H13B	120.00
C8A—C9A—H9A	120.00	C13B—C14B—H14B	120.00
C11A—C10A—H10A	120.00	C15B—C14B—H14B	120.00
C9A—C10A—H10A	120.00	C14B—C15B—H15B	120.00
C6A—C11A—H11A	119.00	C16B—C15B—H15B	120.00
C10A—C11A—H11A	120.00	C15B—C16B—H16B	120.00
C14A—C13A—H13A	119.00	C17B—C16B—H16B	120.00
C12A—C13A—H13A	119.00	C12B—C17B—H17B	120.00
C13A—C14A—H14A	119.00	C16B—C17B—H17B	120.00
C15A—C14A—H14A	119.00	C18B—C19B—H19B	119.00
C16A—C15A—H15A	120.00	C20B—C19B—H19B	119.00
C14A—C15A—H15A	120.00	C19B—C20B—H20B	119.00
C17A—C16A—H16A	122.00	C21B—C20B—H20B	119.00
C15A—C16A—H16A	122.00	C21B—C22B—H22B	119.00
C12A—C17A—H17A	120.00	C23B—C22B—H22B	119.00
C16A—C17A—H17A	120.00	C18B—C23B—H23B	119.00
C18A—C19A—H19A	119.00	C22B—C23B—H23B	119.00
C20A—C19A—H19A	119.00	N24B—C25B—H25D	109.00
C21A—C20A—H20A	119.00	N24B—C25B—H25E	109.00
C19A—C20A—H20A	119.00	N24B—C25B—H25F	109.00
C21A—C22A—H22A	119.00	H25D—C25B—H25E	109.00
C23A—C22A—H22A	119.00	H25D—C25B—H25F	110.00
C22A—C23A—H23A	119.00	H25E—C25B—H25F	109.00
C18A—C23A—H23A	119.00	N24B—C26B—H26D	110.00
H25A—C25A—H25B	110.00	N24B—C26B—H26E	109.00
N24A—C25A—H25C	109.00	N24B—C26B—H26F	109.00
N24A—C25A—H25B	109.00	H26D—C26B—H26E	110.00
H25A—C25A—H25C	109.00	H26D—C26B—H26F	109.00
N24A—C25A—H25A	109.00	H26E—C26B—H26F	109.00
			
C5A—N1A—C2A—N3A	−0.1 (3)	C14A—C15A—C16A—C17A	−6.7 (12)
C5A—N1A—C2A—C18A	179.6 (3)	C15A—C16A—C17A—C12A	4.9 (10)
C2A—N1A—C5A—C4A	0.8 (3)	C2A—C18A—C19A—C20A	178.9 (3)
C2A—N1A—C5A—C6A	−180.0 (3)	C23A—C18A—C19A—C20A	0.1 (5)
C4A—N3A—C2A—N1A	−0.6 (3)	C2A—C18A—C23A—C22A	−179.1 (3)
C4A—N3A—C2A—C18A	179.7 (3)	C19A—C18A—C23A—C22A	−0.3 (5)
C2A—N3A—C4A—C5A	1.0 (3)	C18A—C19A—C20A—C21A	0.3 (5)
C2A—N3A—C4A—C12A	−177.5 (3)	C19A—C20A—C21A—C22A	−0.5 (5)
C25A—N24A—C21A—C20A	0.7 (5)	C19A—C20A—C21A—N24A	−179.6 (3)
C25A—N24A—C21A—C22A	−178.3 (3)	C20A—C21A—C22A—C23A	0.4 (5)
C26A—N24A—C21A—C20A	175.8 (3)	N24A—C21A—C22A—C23A	179.4 (3)
C26A—N24A—C21A—C22A	−3.2 (5)	C21A—C22A—C23A—C18A	0.0 (5)
C2B—N1B—C5B—C6B	173.2 (3)	N1B—C2B—C18B—C19B	−45.5 (4)
C5B—N1B—C2B—N3B	2.0 (3)	N1B—C2B—C18B—C23B	133.4 (3)
C5B—N1B—C2B—C18B	−178.2 (3)	N3B—C2B—C18B—C19B	134.2 (3)
C2B—N1B—C5B—C4B	−2.6 (3)	N3B—C2B—C18B—C23B	−46.9 (4)
C4B—N3B—C2B—C18B	179.8 (3)	N3B—C4B—C5B—N1B	2.4 (3)
C2B—N3B—C4B—C5B	−1.2 (3)	N3B—C4B—C5B—C6B	−172.7 (3)
C2B—N3B—C4B—C12B	174.9 (3)	C12B—C4B—C5B—N1B	−173.3 (3)
C4B—N3B—C2B—N1B	−0.5 (3)	C12B—C4B—C5B—C6B	11.7 (5)
C25B—N24B—C21B—C20B	5.3 (7)	N3B—C4B—C12B—C13B	35.6 (4)
C25B—N24B—C21B—C22B	−175.8 (5)	N3B—C4B—C12B—C17B	−140.6 (3)
C26B—N24B—C21B—C20B	163.9 (5)	C5B—C4B—C12B—C13B	−149.1 (3)
C26B—N24B—C21B—C22B	−17.3 (7)	C5B—C4B—C12B—C17B	34.7 (5)
N1A—C2A—C18A—C23A	176.0 (3)	N1B—C5B—C6B—C7B	43.8 (4)
N3A—C2A—C18A—C19A	176.9 (3)	N1B—C5B—C6B—C11B	−131.1 (3)
N1A—C2A—C18A—C19A	−2.8 (5)	C4B—C5B—C6B—C7B	−141.7 (3)
N3A—C2A—C18A—C23A	−4.3 (5)	C4B—C5B—C6B—C11B	43.3 (5)
N3A—C4A—C12A—C17A	116.5 (4)	C5B—C6B—C7B—C8B	−174.6 (3)
C5A—C4A—C12A—C13A	120.5 (5)	C11B—C6B—C7B—C8B	0.4 (5)
N3A—C4A—C12A—C13A	−61.5 (6)	C5B—C6B—C11B—C10B	174.2 (4)
N3A—C4A—C5A—N1A	−1.1 (4)	C7B—C6B—C11B—C10B	−0.8 (6)
N3A—C4A—C5A—C6A	179.7 (3)	C6B—C7B—C8B—C9B	0.2 (7)
C12A—C4A—C5A—N1A	177.2 (4)	C7B—C8B—C9B—C10B	−0.4 (8)
C12A—C4A—C5A—C6A	−2.0 (6)	C8B—C9B—C10B—C11B	0.0 (9)
C5A—C4A—C12A—C17A	−61.5 (6)	C9B—C10B—C11B—C6B	0.6 (8)
C4A—C5A—C6A—C7A	142.1 (4)	C4B—C12B—C13B—C14B	−178.3 (3)
N1A—C5A—C6A—C7A	−37.0 (5)	C17B—C12B—C13B—C14B	−2.0 (5)
N1A—C5A—C6A—C11A	141.4 (4)	C4B—C12B—C17B—C16B	177.9 (4)
C4A—C5A—C6A—C11A	−39.6 (5)	C13B—C12B—C17B—C16B	1.6 (5)
C5A—C6A—C7A—C8A	−179.8 (4)	C12B—C13B—C14B—C15B	1.4 (6)
C11A—C6A—C7A—C8A	1.8 (6)	C13B—C14B—C15B—C16B	−0.4 (8)
C5A—C6A—C11A—C10A	−178.8 (4)	C14B—C15B—C16B—C17B	0.1 (8)
C7A—C6A—C11A—C10A	−0.4 (6)	C15B—C16B—C17B—C12B	−0.7 (7)
C6A—C7A—C8A—C9A	−2.0 (7)	C2B—C18B—C19B—C20B	176.9 (3)
C7A—C8A—C9A—C10A	0.8 (8)	C23B—C18B—C19B—C20B	−2.1 (5)
C8A—C9A—C10A—C11A	0.6 (9)	C2B—C18B—C23B—C22B	−176.1 (3)
C9A—C10A—C11A—C6A	−0.8 (8)	C19B—C18B—C23B—C22B	2.9 (5)
C17A—C12A—C13A—C14A	0.6 (8)	C18B—C19B—C20B—C21B	−0.5 (6)
C4A—C12A—C17A—C16A	179.9 (5)	C19B—C20B—C21B—N24B	−178.8 (4)
C4A—C12A—C13A—C14A	178.7 (5)	C19B—C20B—C21B—C22B	2.3 (6)
C13A—C12A—C17A—C16A	−2.0 (8)	N24B—C21B—C22B—C23B	179.6 (4)
C12A—C13A—C14A—C15A	−2.6 (11)	C20B—C21B—C22B—C23B	−1.5 (6)
C13A—C14A—C15A—C16A	5.6 (13)	C21B—C22B—C23B—C18B	−1.1 (6)

### Hydrogen-bond geometry (Å, º)

**Table:**

*D*—H···*A*	*D*—H	H···*A*	*D*···*A*	*D*—H···*A*
N1*B*—H1*B*···N1*A*^i^	1.01	1.92	2.899 (3)	163
N3*A*—H3*A*···N3*B*	1.02	1.92	2.890 (3)	157
C19*A*—H19*A*···N1*A*	0.93	2.63	2.943 (4)	100

Symmetry code: (i) *x*−1/2, −*y*+3/2, *z*−1/2.
